# A case of chronic asymptomatic central pontine myelinolysis with histological evidence of remyelination

**DOI:** 10.1007/s12024-017-9933-y

**Published:** 2017-11-25

**Authors:** Harry R. Haynes, Patrick J. Gallagher, Andrea Cordaro, Marcus Likeman, Seth Love

**Affiliations:** 1 Department of Cellular Pathology, North Bristol NHS Trust, Bristol, UK; 2 University of Bristol Medical School, Bristol, UK; 3 Department of Neuroradiology, North Bristol NHS Trust, Bristol, UK; 4 Department of Neuropathology, North Bristol NHS Trust, Bristol, UK

**Keywords:** Central pontine myelinolysis, Remyelination, Post-mortem, Alcohol, Asymptomatic

## Abstract

Central pontine myelinolysis (CPM) is a neurological demyelinating disease of the pons. Although usually associated with rapid correction of hyponatremia, CPM may occur despite normonatremia, is often associated with chronic alcoholism and may be asymptomatic. Histological confirmation of asymptomatic CPM is rare. We describe an unusual post-mortem case of extensive but asymptomatic CPM in a chronic alcoholic patient with normonatremia. The affected part of the pons contained thinly myelinated axons with appearances supporting remyelination. We suggest that remyelination may account for the subclinical nature of this patient's CPM.

## Introduction

Central pontine myelinolysis (CPM) is a non-inflammatory demyelinating disease of the pons, first described in 1959 [1]. Extrapontine myelinolysis may additionally occur in the basal ganglia or cerebellum. Macroscopically, bilateral symmetrical softening and discoloration is seen in the base of the pons. Histology reveals loss of myelin and oligodendroglia with relative sparing of axons and neurons [2]. The clinical severity ranges from an asymptomatic finding on neuroimaging to locked-in syndrome, coma and death [2]. CPM may present with dysarthria, dysphagia, hyperreflexia or paralysis. The extent of the demyelination has not been found to correlate with the symptoms.

## Clinical summary

A 47-year-old chronic alcoholic self-discharged from hospital where he was being treated for alcohol withdrawal. No focal neurological abnormalities had been noted on examination and serum electrolytes were within normal limits. 72 h later he was found deceased in a wooded area, partially undressed.

At autopsy, the stomach lining contained numerous black areas (‘Wischnewski spots’), supportive of a diagnosis of fatal hypothermia. The ‘paradoxical undressing’ described above is a well-recognized feature of deaths secondary to hypothermia (defined by a fall in core body temperature to below 35 °C).

**Fig. 1:**
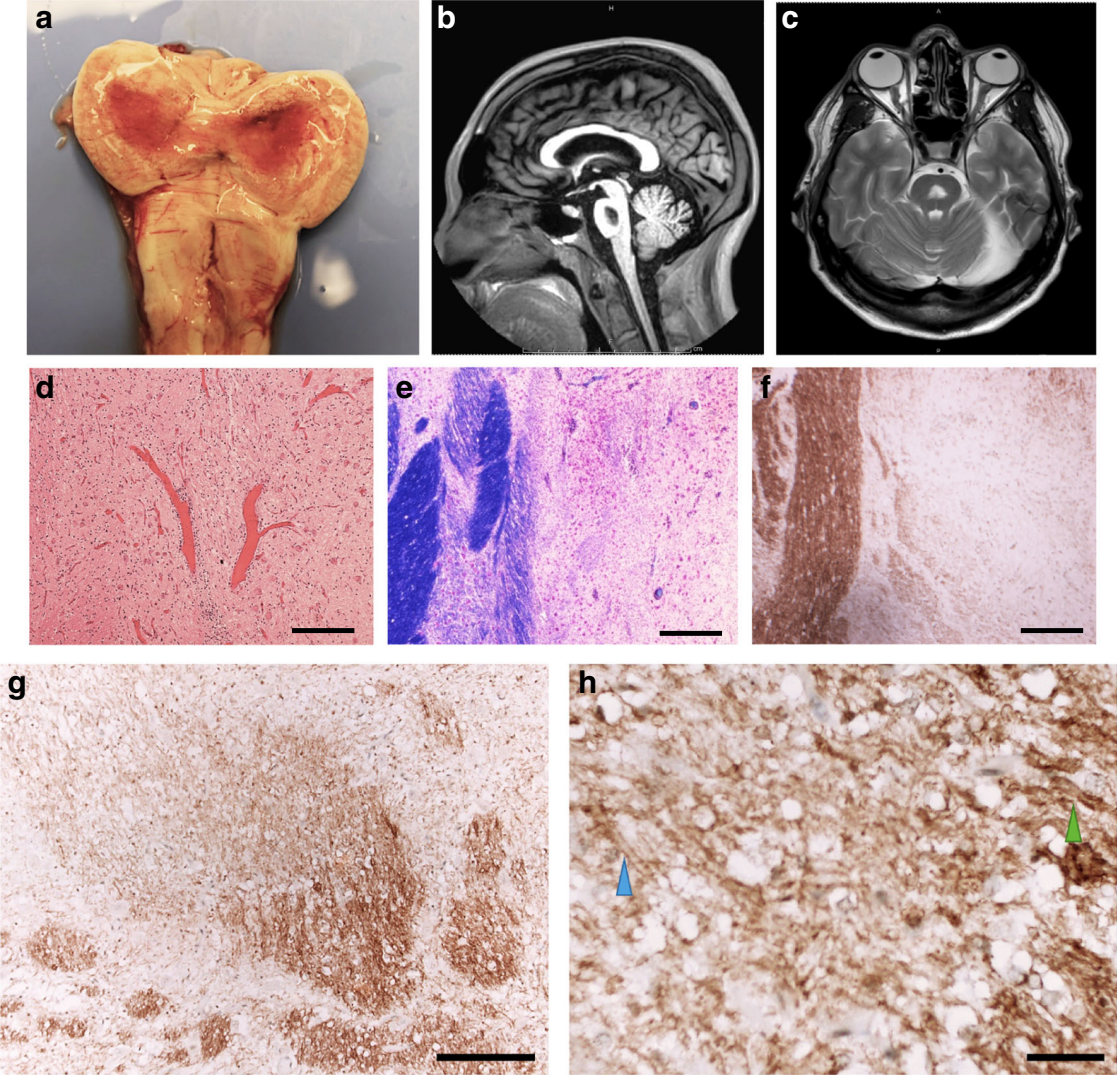
**a** Post-mortem image of the fresh pons and medulla, opened sagitally from the ventral aspect. A red, central, triangular region of softening is seen with preservation of the surrounding parenchyma. **b** Sagittal T1 and (**c**) Axial T2 ante-mortem MRI sequences. The pons includes a trident-shaped focus of high T2 signal. Cerebellar atrophy is also noted, in keeping with the history of alcoholism. **d** Hypercellular pontine tissue, reflecting astrocytic gliosis and perivascular and parenchymal accumulation of chronic inflammatory cells (scale bar = 200 μm). **e** Staining with luxol fast blue and cresyl violet revealed a sharply demarcated region of reduced staining of myelin in the central part of the base of the pons, with good preservation of neurons (scale bar = 200 μm). **f & g** There was reduced labelling of myelin with anti-PLP1 in the affected region of pons (scale bar = 200μm). **h** Included within this region were many large-caliber but thinly myelinated axons (blue arrowhead), in contrast to the normally myelinated, large-caliber fibers in the adjacent pons (green arrowhead) (scale bar = 50μm)

Examination of the brain revealed a symmetrical, central region of discoloration and partial cavitation within the pons, up to 25 mm across (Fig. 1a). A corresponding lesion had been seen on an ante-mortem MRI scan two years previously (Fig. 1b & c). No further white matter changes were seen macroscopically, including within the corona radiata, internal capsule, basal ganglia or cerebellar white matter.

## Pathological findings

Histology showed demyelination with astrocytosis and infiltration by lymphocytes and macrophages (Fig. 1d). Staining with luxol fast blue and cresyl violet revealed a sharply demarcated region of demyelination in the central part of the base of the pons, with good preservation of neurons (Fig. 1e). Anti-myelin PLP1 immunohistochemistry (IHC) confirmed the loss of myelin (Fig. 1f & g).The appearance was typical of central pontine myelinolyis (CPM). However, included within the region of demyelination were many thin myelin sheaths that were weakly but convincingly immunopositive for PLP1. This pattern of immunopositivity suggested reparative myelination had occurred (Fig. 1h).

## Discussion

The main cause of CPM is the rapid correction of hyponatremia [3] exposing pontine oligodendrocytes to osmotic stress [4]. This most often occurs in the context of alcoholic liver disease. It has been proposed that osmotic injury to vascular endothelial cells after the rapid reversal of hyponatremia releases myelinotoxic factors [5]. This causes vasogenic edema and demyelination at highly vascular grey-white matter interfaces such as the pons [5]. CPM also occurs in normonatremia, with the majority of cases associated with chronic alcoholism. Alcoholism may deplete intracellular glucose and glycogen, resulting in metabolic stress and a pro-apoptotic milieu, in which excess production of free radicals and nitric oxide leads to myelin injury [6].

In this case, serum sodium was normal ante-mortem and the patient was asymptomatic with no previous history of neurological disease. An autopsy series of over 3000 patients found 15 cases of CPM, all of which were asymptomatic (12 out of the 15 were associated with alcoholism). The majority of active lesions were associated with a rapid reversal of hyponatremia [7]. Asymptomatic CPM has been reported in the absence of documented electrolyte disturbance [8], although histological confirmation of the lesion, as seen in this case, is extremely rare.

It is increasingly recognized that functional neurological recovery is possible after a diagnosis of CPM [9]. Although serial MRI studies have shown that radiological resolution does occur [10], the temporal evolution of radiological features is not considered to have prognostic value in predicting clinical outcome [11]. In this case, histological features of remyelination were seen within the lesion which, to the authors’ knowledge, have not previously been demonstrated. Remyelination is likely to underpin neurological and radiological improvement after acute osmotic demyelination, and we suggest that remyelination could explain why the present patient was asymptomatic prior to his death. Improved understanding of the process of remyelination after CPM may help to identify radiological markers that better predict clinical outcome and guide the development of therapies.

## Key points

1. Chronic alcohol abuse is a risk factor for the development of CPM, which may occur with normonatremia.
2. Neurological recovery has been reported after CPM and we postulate that remyelination may account for this.
3. Further research into the histological and radiological markers of remyelination in CPM is needed.

## References

[1] Adams RD, Victor M, Mancall EL (1959). Central pontine myelinolysis: a hitherto undescribed disease occurring in alcoholic and malnourished patients. AMA Arch Neurol Psychiatry.

[2] Hurley RA, Filley CM, Taber KH (2011). Central pontine myelinolysis: a metabolic disorder of myelin. J Neuropsychiatry Clin Neurosci.

[3] Kleinschmidt-DeMasters BK, Norenberg MD (1981). Rapid correction of hyponatremia causes demyelination: relation to central pontine myelinolysis. Science.

[4] Adrogué HJ, Madias NE (2000). Hyponatremia. N Engl J Med.

[5] Norenberg MD (1983). A hypothesis of osmotic endothelial injury. A pathogenetic mechanism in central pontine myelinolysis. Arch Neurol.

[6] Ashrafian H, Davey P (2001). A review of the causes of central pontine myelinosis: yet another apoptotic illness?. Eur J Neurol.

[7] Newell KL, Kleinschmidt-DeMasters BK (1996). Central pontine myelinolysis at autopsy; a twelve year retrospective analysis. J Neurol Sci.

[8] Razvi SS, Leach JP (2006). Asymptomatic pontine myelinolysis. Eur J Neurol.

[9] Louis G, Megarbane B, Lavoué S, Lassalle V, Argaud L, Poussel JF (2012). Long-term outcome of patients hospitalized in intensive care units with central or extrapontine myelinolysis. Crit Care Med.

[10] Buis C (2002). Serial magnetic resonance imaging of central pontine myelinolysis. Liver Transplant.

[11] Beh SC (2017). Temporal evolution of the trident and piglet signs of osmotic demyelination syndrome. J Neurol Sci.

